# Role of the flagellar hook in the structural development and antibiotic tolerance of *Pseudomonas aeruginosa* biofilms

**DOI:** 10.1038/s41396-021-01157-9

**Published:** 2021-12-08

**Authors:** Jules D. P. Valentin, Hervé Straub, Franziska Pietsch, Marion Lemare, Christian H. Ahrens, Frank Schreiber, Jeremy S. Webb, Henny C. van der Mei, Qun Ren

**Affiliations:** 1 Laboratory for Biointerfaces, Empa, The Swiss Federal Laboratories for Materials Science and Technology, 9014 St. Gallen, Switzerland; 2 Department of Biomedical Engineering, University of Groningen and University Medical Center Groningen, 9712 CP Groningen, the Netherlands; 3 Department of Plant and Microbial Biology, University of Zürich, 8008 Zürich, Switzerland; 4 Division of Biodeterioration and Reference Organisms, Department of Materials and the Environment, Federal Institute for Materials Research and Testing (BAM), 12205 Berlin, Germany; 5 Agroscope, Research Group Molecular Diagnostics, Genomics & Bioinformatics and SIB Swiss Institute of Bioinformatics, 8820 Wädenswil, Switzerland; 6 National Biofilms Innovation Centre, Institute for Life Sciences, University of Southampton, Southampton, SO16 7 PX UK

**Keywords:** Microbiology, Diseases

## Abstract

*Pseudomonas aeruginosa* biofilms exhibit an intrinsic resistance to antibiotics and constitute a considerable clinical threat. In cystic fibrosis, a common feature of biofilms formed by *P. aeruginosa* in the airway is the occurrence of mutants deficient in flagellar motility. This study investigates the impact of flagellum deletion on the structure and antibiotic tolerance of *P. aeruginosa* biofilms, and highlights a role for the flagellum in adaptation and cell survival during biofilm development. Mutations in the flagellar hook protein FlgE influence greatly *P. aeruginosa* biofilm structuring and antibiotic tolerance. Phenotypic analysis of the *flgE* knockout mutant compared to the wild type (WT) reveal increased fitness under planktonic conditions, reduced initial adhesion but enhanced formation of microcolony aggregates in a microfluidic environment, and decreased expression of genes involved in exopolysaccharide formation. Biofilm cells of the *flgE* knock-out mutant display enhanced tolerance towards multiple antibiotics, whereas its planktonic cells show similar resistance to the WT. Confocal microscopy of biofilms demonstrates that gentamicin does not affect the viability of cells located in the inner part of the *flgE* knock-out mutant biofilms due to reduced penetration. These findings suggest that deficiency in flagellar proteins like FlgE in biofilms and in cystic fibrosis infections represent phenotypic and evolutionary adaptations that alter the structure of *P. aeruginosa* biofilms conferring increased antibiotic tolerance.

## Introduction

Infections caused by the opportunistic pathogen *Pseudomonas aeruginosa* are among the most prevalent and difficult to treat [1, 2]. Besides its intrinsic antibiotic resistance, *P. aeruginosa* often grows as a community of cells aggregated within an extracellular matrix, called a biofilm [3]. Biofilms are known to promote the survival of bacteria under harsh environmental conditions, and to protect bacterial cells from immune responses and antibiotic treatment [4, 5]. The biofilm including the matrix is one of the factors leading to this recalcitrance, providing a barrier against antibiotics and allowing different metabolic states within the biofilm population [6–8]. *P. aeruginosa* biofilms can display complex and 3-dimensional architectures, including suspended aggregates which have been observed in clinical samples [9, 10], and mushroom-like structures that are commonly observed in laboratory model systems [11–13]. Known to be influenced by chemotaxis [14], bacterial motility [15], and environmental parameters [12, 16, 17], the stalks of mushroom-like structures were shown to be initiated by proliferation of non-motile bacteria, while the caps were formed after climbing and aggregation of a motile subpopulation [15]. Although twitching was first considered to have the most critical role for the formation of the mushroom cap [15], flagellum-driven surface-associated motility was shown to play an equally important role [18]. A better understanding of the contribution of biofilm structure to bacterial survival may lead to improved strategies for biofilm control and antibiotic treatment.

Despite its importance, the flagellum is known to be immunogenic [19]. *P. aeruginosa* downregulates flagellum synthesis during biofilm maturation and upon contact with cystic fibrosis (CF) airway liquid [20, 21]. In fact, *P. aeruginosa* CF isolates are often non-flagellated and showed high variability in motility and in biofilm formation [22, 23]. By comparing the transcriptomes of biofilm and planktonic cells, a narrower set of genes involved in pili and flagella synthesis was found to be repressed in *P. aeruginosa* biofilms [24]. Among the repressed genes involved in motility, the *flgE* (PA1080) gene encodes for the flagellar hook, a curved structure at the basis of the flagellum [25]. The flagellar hook protein FlgE is known to be crucial for flagellar synthesis and swimming motility [26, 27]. *FlgE* mutants were also found to be more readily permeabilized and killed by surfactant protein A than the wild type (WT) [28]. However, FlgE is known to be pro-inflammatory [29, 30] and has been found to be absent from the membranes of *P. aeruginosa* PAK cells grown in mucus [31].

Although *flgE* has been reported to be involved in motility and CF adaptation, its overall impact on bacterial adhesion, biofilm formation and resistance has not been systematically studied. This study reports for the first time that inactivating the *flgE* gene promotes the emergence of aggregated structures in *P. aeruginosa* biofilms, decreases the expression of biofilm matrix genes, and drastically increases the biofilm-associated tolerance to antibiotics. Lacking swimming ability, the *flgE* mutant exhibits a reduced ability to adhere to surfaces, but a faster biofilm growth once adherent as compared to the WT. While the planktonic resistance remains unchanged, inactivating the *flgE* gene increases tolerance of biofilm cells towards multiple antibiotics, such as gentamicin and colistin. Observations by confocal laser scanning microscopy reveal that gentamicin did not affect cells located in the inner part of the biofilm, which is linked to the reduced antibiotic penetration. This study shows that repression of the *flgE* gene in biofilms and likely also in CF lung infections alters the structure of *P. aeruginosa* biofilms, promoting bacterial antibiotic tolerance.

## Materials and methods

If not stated differently, all chemicals and reagents were purchased from Sigma-Aldrich (Buchs, Switzerland).

### Bacterial strains and culture media

*P. aeruginosa* MPAO1 WT and transposon mutants (*arnB*, *fiuA*, *flgD* and *flgE*) were purchased from the Manoil Lab [32]. *Escherichia coli* DH5α containing the plasmid pK19mobsacB and *E. coli* St18 suspensions were prepared in Lennox Broth (LB, Roth, Germany) media for plasmid amplification and conjugation with *P. aeruginosa*, respectively [33]. Overnight cultures of *P. aeruginosa* were prepared in Brain Heart Infusion (BHI, CM1135, Oxoid, UK) medium at 37 °C at 180 rpm. Biofilm formation and antibiotic assays were performed in M9 medium (M9 minimal salts 5×, M6030, USA) containing 48 mM Na_2_HPO_4_, 22 mM KH_2_PO_4_, 9 mM NaCl, 19 mM NH_4_Cl, 2 mM MgSO_4_ (63140-F, Japan) 100 µM CaCl_2_ (21100, Fluka, Germany) and 20 mM glucose (G7528, USA) [34].

### Construction of knockout mutants

The genomic knockout of the *flgE* gene in *P. aeruginosa* MPAO1 was performed by a two-step allelic exchange leading to an unmarked deletion, as described by Huang et al. [35]. All primers used in this study were ordered from Microsynth. The vector pK19mobsacB was assembled with the upstream and the downstream regions of the *flgE* gene using NEBuilder HiFi DNA Assembly Master Mix (E2621, NEB, USA) and two sets of primers, *flgE*-frag1 and *flgE*-frag2 (Table S1). Pk19mobsacB plasmid was a suicide vector in *P. aeruginosa* MPAO1 carrying two markers, kanamycin resistance and sucrose sensitivity. The following transformations were performed by heat shock at 42 °C for 2 min. The assembled vector was transformed to *E. coli* DH5α for amplification and the constructions were confirmed by PCR (T3000 Thermocycler, Biometra, Germany) as detailed in the following paragraph. Next, the vector was transformed to *E. coli* St18, auxotrophic for aminolevulinic acid, with selection on LB agar (05039, Spain) plates containing 50 µg/mL of aminolevulinic acid (A3785, Israel) and 25 µg/mL of kanamycin (T832.3, Carl Roth, Germany). The first homologous recombination leading to the integration of Pk19mobsac in *P. aeruginosa* MPAO1 was obtained through conjugation between *P. aeruginosa* MPAO1 and *E. coli* St18 + Pk19mobsacB. Bacterial cultures of *P. aeruginosa* MPAO1 and *E. coli* St18 were mixed 1:1 and grown on LB agar plates containing 50 µg/mL of aminolevulinic acid. After 24 h, the lawns were scraped, diluted and spread on LB agar plates containing 200 µg/mL of kanamycin to select *P. aeruginosa* MPAO1 cells with genomic integration of Pk19mobsac. The second homologous recombination leading to the excision of pK19mobsac, alongside with the excision of the targeted gene, was obtained after culture in LB media without selection marker and then selection on LB agar plates containing 10% sucrose (84097, Switzerland) and no NaCl. Transformants able to grow in the presence of sucrose were determined to be kanamycin sensitive, confirming the removal of pK19mobsacB. The knockout mutant generated here can be made available upon request.

### Confirmation of gene deletion

Gene deletion was confirmed through PCR amplification of the deletion site and consequent sequencing. To confirm the recombination events during knocking out procedure, bacterial colonies grown on LB agar plates were suspended in 100 µL of ultrapure water to perform colony PCR. For the deletion of the *flgE* gene, the primers *flgE*-Frag1-Fw and *flgE*-Frag2-Rv were used to amplify an amplicon of 2459 bp or 1070 bp in presence or absence of the *flgE* gene, respectively. All PCR analysis were performed according to the protocol detailed in the supplementary materials. Sequencing of the deletion site of MPAO1 Δ*flgE* confirmed that the targeted gene was removed and the upstream and downstream regions were bound together without any residual nucleotides.

### Biofilm cells tolerance

Our screening protocol was adapted from Mah et al. [36] and has been described in detail in our previous work [37]. Overnight cultures of *P. aeruginosa* MPAO1 and mutants prepared in BHI media were diluted 1:100 in M9 medium. Biofilm formation was performed in 96-well plates during 24 h incubation at 37 °C. Biofilm biomass was quantified by crystal violet staining (0.1%, 30 min, 61135, Fluka, India). To measure biofilm tolerance, biofilms were washed with 0.9% NaCl (71380, USA) to remove planktonic cells and incubated for 24 h at 37 °C in M9 medium supplemented with either colistin (C4461, China) or gentamicin (G1914, USA). Biofilms treated with antibiotics were washed with 0.9% NaCl and biofilm cells that may have survived were allowed to recover in antibiotic-free M9 medium for 24 h at 37 °C. The turbidity of the medium suspension was measured spectrophotometrically at optical densities 600 nm (OD600, Genesys 6, USA) after 24 h to evaluate the presence of survivors. In a second step, the number of viable cells in the biofilm suspension after treatment and recovery was assessed by plate counting of the colony forming units (CFU) or by spotting 5 µL on BHI agar plates to determine the minimal bactericidal concentrations for biofilms (MBC-B).

### Planktonic cells tolerance

The minimal bactericidal concentration for planktonic cells (MBC-P) of *P. aeruginosa* MPAO1 WT and Δ*flgE* mutant was assessed in 96-well plates, as described by Mah [36]. Following the same bacterial culture preparation as described above, M9 media supplemented with a gradient of concentration of gentamicin, tobramycin, colistin, ciprofloxacin, meropenem, or imipenem were inoculated with MPAO1 and mutants at a final concentration of 5 × 10^6^ CFU/mL. The bacterial growth was followed by measuring the OD600 during 24 h incubation at 37 °C. After 24 h of treatment, 5 µL of the bacterial suspension was spotted on BHI agar plates and MBC-P values were determined after 24 h incubation. The MBC-P and MBC-B are defined as the lowest concentration of a drug resulting in at least 3-log reduction of a planktonic and biofilm population, respectively.

### Bacterial motility

The swimming assay was performed according to the protocol described by Déziel et al. [38]. Swimming plates were prepared with 1% tryptone (16922, Fluka, Germany), 0.5% NaCl and 0.3% agar. Bacterial cultures prepared in M9 medium at 5 × 10^6^ CFU/mL were stabbed in the agar with a sterile toothpick and the spreading was quantified with ImageJ after 24 h incubation at 37 °C.

### Bacterial adhesion, biofilm formation, and antibiotic treatment under flow

*P. aeruginosa* strains were observed under flow using a custom-made microfluidic device (Fig. S1) [39]. Briefly, a microfluidic flow chamber (length 24 mm, width 3 mm, height 320 µm) made of PDMS with three inlet channels (each of 12 mm length, width 1 mm, height 320 µm) was bonded on glass. This microfluidic flow chamber was mounted on an automated inverted microscope (Ti2-E, Nikon, Japan) with 10× (CFI Plan Fluor, N.A. 0.3, Japan) and 40× (CFI S Plan Fluor, N.A. 0.6, Japan) air objectives. The flow was controlled and monitored by a pressure controller and a set of flow sensors (OB1, Elveflow, France). The experiments were performed at room temperature in three consecutive phases, namely, (1) preconditioning and bacterial inoculation, (2) biofilm growth, and (3) antibiotic treatment. (1) The flow chamber was preconditioned by flowing M9 medium for 30 min followed by the injection of bacterial suspension at a concentration of 5 × 10^6^ CFU/mL through the middle channel for 1 h at 200 µL/min. Bacterial adhesion on glass was monitored by recording ten positions in the middle of the chamber in bright field mode every 10 min and then quantified by automated counting with the software ImageJ. (2) The flow of bacterial suspension was stopped and sterile M9 medium was injected through the top and bottom channels over 40 h at 200 µL/min. Biofilm growth was quantified by measuring the surface covered by bacteria over time in ten locations in the middle channel. (3) M9 medium supplemented with 3 µM of propidium iodide (PI, Live/Dead BacLight Bacterial Viability Kit, L7007, Invitrogen, USA) and 12 µg/mL of gentamicin was injected through the top channel at 100 µL/min while M9 medium supplemented only with 3 µM of PI was injected through the bottom channel at 100 µL/min. Due to the stable laminar flow regime in the chamber, the two different streams did not mix across the length of the chamber. Nevertheless, biofilm cells at the interface of the two flows were not considered in order to rule out the diffusion zone of gentamicin in the antibiotic free stream. The concentration of PI used here has been shown to not negatively affect bacteria [40]. Biofilm cells during treatment were observed in bright field and with red fluorescence. After 24 h of treatment, biofilm cells were stained with live dead staining using 3 µM of SYTO9 (Live/Dead BacLight Bacterial Viability Kit, L7007, Invitrogen, USA) and 20 µM of PI followed by confocal laser scanning microscopy (LSM780, Zeiss, Germany) observations (objective LD Plan-Neofluar 20×/0.4 Korr, excitation wavelengths of 488 and 561 nm). The images were analyzed with ImageJ.

### Gentamicin penetration through the biofilm matrix

Biofilms of MPAO1 WT and Δ*flgE* mutant were prepared as described above on polycarbonate slides in 96-well plates during 72 h with renewal of the medium every 24 h. Biofilms cells were then stained with 3 µM of SYTO9 and treated with 2 µg/mL of Texas Red fluorophore conjugated gentamicin (TR-gentamicin, Ursa BioScience) for 20 min. After washing with 0.9% NaCl, the treated biofilms were observed by confocal laser scanning microscopy (objective C-Apochromat 40×/1.20 W Korr M27, excitation wavelengths of 488 and 561 nm).

### Quantification of mRNA levels of matrix genes in biofilms

Biofilms of MPAO1 WT and Δ*flgE* mutant were prepared as described above in 96-well plates during 24 h incubation at 37 °C. RNA of biofilms was extracted with the RNAprotect Bacterial Reagent kit (Qiagen, 75606), and genomic DNA was eliminated with the TURBO DNA-free kit (Invitrogen, AM1907). Total RNA was reversely transcribed to the complementary DNA using the iScript cDNA synthesis kit (BioRad, 1708891). Primers specific to the housekeeping gene, *rpsL*, and the biofilm matrix genes, *pelA*, *pelB*, *pslA*, *pslB*, and *cdrA*, in *P. aeruginosa* were designed and are listed in Table S1. Quantitative PCR (qPCR) analysis was performed with the iQ SYBR Green Supermix kit (BioRad, 1708880) according to the manufacturer’s instructions. The cycling condition was composed of an initial denaturation of 10 min at 95 °C, followed by 40 cycles of 10 s at 95 °C, 60 s at 60 °C, and 15 s at 72 °C. The 2^−ΔΔCt^ method was used to measure the fold change in gene expression compared to the housekeeping gene *rpsL* and the results were normalized to the expression levels of the WT for each gene [41].

### Scanning electron microscopy (SEM)

SEM images were taken using a field-emission SEM (Zeiss LEO1550) at 1 kV under 20k×, 30k×, and 50k× magnification with stage angles of 0° and 30°.

## Results

### FlgE plays a greater role in biofilm formation and biofilm tolerance than FlgD

**Fig. 1:**
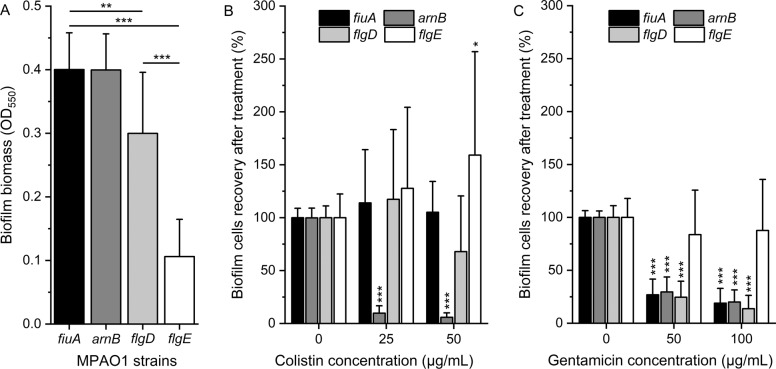
Biofilm formation of MPAO1 transposon mutants and their tolerance towards colistin and gentamicin. **A** The biomass of 24 h-old biofilms of *P. aeruginosa* MPAO1 transposon mutants grown in M9 medium in 96-well plates was quantified by crystal violet staining. Results represent the mean ± SD of two biological repeats with eight technical repeats each. Student’s *t*-tests were performed with ** equals to *P* < 0.01 and *** equals to *P* < 0.001. Tolerance of biofilm cells to **B** colistin and **C** gentamicin was assessed by measuring the optical density of the supernatant after the recovery of treated biofilm cells compared to non-treated ones (defined as 100%). Results represent the mean ± SD of two biological repeats (three for *arnB* and *flgE* mutants) with four technical repeats each. Student’s *t*-tests were performed in comparison to the condition without antibiotics for each mutant with * equals to *P* < 0.05 and *** equals to *P* < 0.001.

To investigate the role of the flagellar hook on biofilm formation, transposon mutants of *P. aeruginosa flgD* (strain PW2945, PA1079-E05::ISlacZ/hah) and *flgE* (strain PW2947, PA1080-C11:: ISphoA/hah) genes, constructed by the Manoil Lab [32], were analyzed (Fig. 1A). The transposon mutants *fiuA* (strain PW1861, PA0470-F05:: ISlacZ/hah) and *arnB* (strain PW7021, PA3552-G12:: ISphoA/hah) were selected as reference strains for biofilm formation instead of *P. aeruginosa* MPAO1 WT, to eliminate the bias caused by the presence of the Tn5 transposon [37]. The *fiuA* gene product is involved in the transport of the heterologous siderophore ferrichrome [42]. Ferrichromes are produced by fungi and not present in our experiments, making the *fiuA* mutant a suitable reference. The *arnB* gene product is involved in resistance to colistin but not to gentamicin [43, 44]. Inactivation of the *arnB* gene in *P. aeruginosa* was shown to have no effect on biofilm formation [37].

Inactivating the genes encoding the flagellar basal-body rod modification protein FlgD and the flagellar hook protein FlgE reduced the biofilm biomass by 25% and 73% compared to references (Fig. 1A), respectively, suggesting an important role of *flgE* in biofilm formation. By adapting the protocol developed by Mah [36] we measured the ability of biofilm cells to recover from antibiotic treatment. The mutant *arnB* showed a recovery of 10 and 6% of biofilm cells after exposure to 25 and 50 µg/mL of colistin, respectively, compared to the untreated cells (Fig. 1B). In comparison, inactivating the *fiuA*, *flgD*, and *flgE* genes had no influence on the resistance of *P. aeruginosa* biofilm cells towards colistin at 25 µg/mL. At 50 µg/mL colistin, the recovery of the *flgD* mutant was reduced to 68%, whereas the *flgE* mutant showed an increased growth compared to the untreated biofilm. Biofilm cells of *fiuA, arnB*, and *flgD* mutants were found to be more sensitive to gentamicin, with 70 and 80% reduction in recovery after 50 and 100 µg/mL, respectively (Fig. 1C). In contrast, biofilm cells of the *flgE* mutant were able to recover as well as the non-treated biofilm even after exposure to 100 µg/mL of gentamicin. These results demonstrate that the inactivation of the *flgE* gene led to a major change in biofilm physiology and survival of *P. aeruginosa* following antibiotic treatment.

### *FlgE* mutants exhibit enhanced and biofilm-specific tolerance to gentamicin, colistin, and ciprofloxacin

**Fig. 2:**
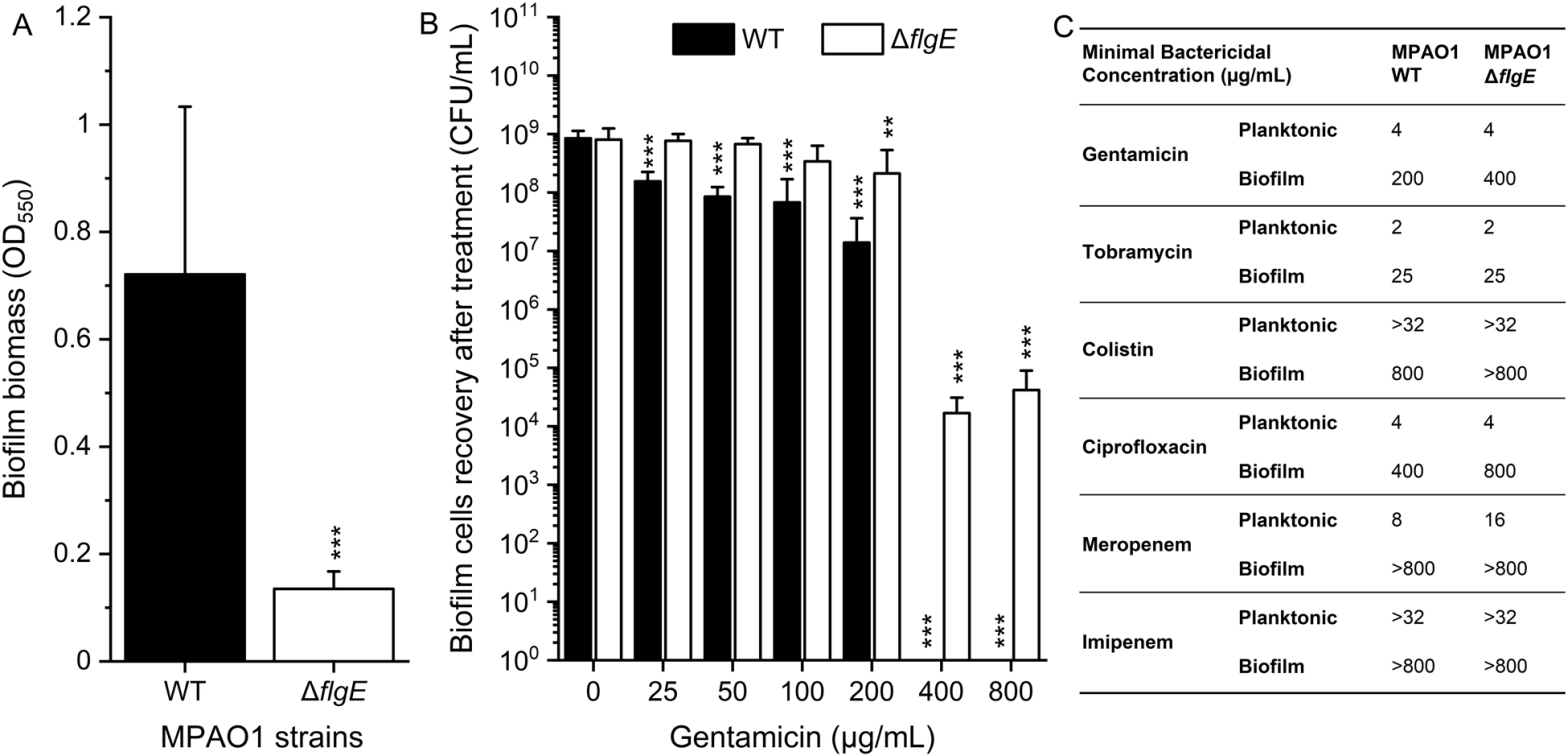
Biofilm formation, biofilm tolerance, and planktonic resistance of MPAO1 Δ*flgE* towards different antibiotics. **A** The biomass of the 24 h-old biofilms of *P. aeruginosa* MPAO1 WT and ΔflgE grown in M9 medium in 96-well plates was quantified by crystal violet. Results represent the mean ± SD of three biological repeats with eight technical repeats each. Student’s *t*-tests were performed with *** equals to *P* < 0.001. **B** Twenty-four hours-old biofilms were exposed to M9 medium supplemented with a gradient of gentamicin concentrations for 24 h and CFUs were counted after 24 h recovery in antibiotic-free M9 medium. Results represent the mean ± SD of three biological repeats with two technical repeats each. Student’s *t*-tests were performed in comparison to the untreated strain population with **P < 0.01 and ***P < 0.001. **C** The resistance profile of MPAO1 Δ*flgE* was measured by quantifying the minimal bactericidal concentrations of various antibiotics towards planktonic and biofilm cells population. The MBC-P and MBC-B are defined as the lowest concentration of a drug resulting in at least 3-log reduction of a planktonic and biofilm population, respectively. The MBC-P were measured by spotting a planktonic population on BHI agar after 24 h treatment and the MBC-B by spotting the biofilm suspension after 24 h treatment and 24 h recovery (three biological repeats with two technical repeats each, where the most representative maximal values are shown).

To improve our understanding of the increased tolerance, we deleted the *flgE* gene in *P. aeruginosa* MPAO1 WT by genomic knockout and assessed its importance for both biofilm and planktonic resistance. The complete in-frame removal of the *flgE* was confirmed by PCR and sequencing (Fig. S2). Similar to the *flgE* transposon mutant, in-frame deletion of the *flgE* gene led to a decrease of 81% in biofilm biomass compared to the MPAO1 WT (Fig. 2A). The recovery of the Δ*flgE* mutant biofilm was not statistically significantly affected by gentamicin up to 100 µg/mL of, while biofilm cells of MPAO1 WT exhibited significantly reduced recovery after 25 µg/mL of gentamicin (Fig. 2B). Exposure to 200 µg/mL of gentamicin led to an approximately 2-log reduction in the recovery of MPAO1 WT biofilm cells, while 400 and 800 µg/mL led to a complete elimination of the WT biofilm cells. After treatment with 400 and 800 µg/mL of gentamicin, biofilm cells of the Δ*flgE* mutant were still able to recover up to 10^4^ CFU/mL. Complementing the *flgE* gene in the Δ*flgE* mutants restored the sensitive phenotype of the WT, confirming the importance of this gene (Fig. S3). Different types of antibiotics were measured for their MBC-P and MBC-B following Mah’s protocol (Fig. 2C) [36]. The MBC-P and MBC-B are defined as the lowest concentration of a drug resulting in at least 3-log reduction of a planktonic and biofilm population, respectively. Planktonic cells of the Δ*flgE* mutant were found to show similar resistance to the WT towards most tested antibiotics, except for meropenem. Consistent with our previous results, biofilm cells of the Δ*flgE* mutant exhibited increased tolerance to gentamicin (MBC-B of 200 and 400 µg/mL for WT and Δ*flgE* mutant biofilms, respectively). In addition, the Δ*flgE* mutant exhibited an increased biofilm-specific tolerance towards colistin and ciprofloxacin, but similar tolerance towards tobramycin, meropenem and imipenem as compared to the WT. Thus, the absence of *flgE* in *P. aeruginosa* conferred a biofilm-specific mechanism of tolerance allowing the survival of biofilm cells in the presence of several antibiotics.

### *FlgE* mutants exhibit reduced swimming and enhanced planktonic fitness

**Fig. 3:**
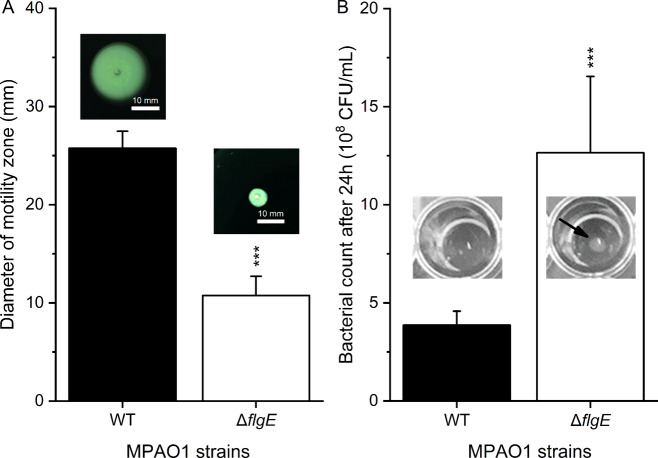
Phenotypes of MPAO1 WT and Δ*flgE* with respect to (A) swimming and (B) bacterial growth. **A** Diameters of bacterial spreading on 0.3% swim agar were measured after 24 h of growth at 37 °C. Representative images of the swimming agar plates are shown above each bar. Results represent the mean ± SD of four biological repeats with two measurement per repeat. Student’s *t*-test was performed in comparison to the WT with *** equals to *P* < 0.001. **B** Bacterial suspensions were grown for 24 h in M9 medium at 37 °C under static conditions. Representative pictures of WT and Δ*flgE* wells are shown and the CFU/mL were measured. Results represent the mean ± SD of four biological repeats with two technical repeats each. A Student’s *t*-test was performed in comparison to the WT with ***P < 0.001. The arrow indicates the sedimentation of Δ*flgE* mutant cells during static incubation.

We further assessed whether the low biofilm formation of the Δ*flgE* mutant was related to bacterial motility. To this end, swimming ability was quantified by measuring the spreading of *P. aeruginosa* on swim agar plates. The Δ*flgE* mutant exhibited an average diameter of spreading of 11 ± 2 mm, much lower than the 26 ± 2 mm of MPAO1 WT (Fig. 3A). The Δ*flgE* mutant was further investigated with respect to its relative fitness measured as growth yield and growth kinetics during static incubation. The planktonic Δ*flgE* mutant exhibited a higher growth rate than the MPAO1 WT (Fig. S4) and an approximately three times higher cell density after 24 h in M9 medium (Fig. 3B), suggesting an enhanced growth yield. Additionally, an agglomerate of Δ*flgE* planktonic cells was observed at the bottom of the well after 24 h growth, which is a characteristic of non-flagellated bacteria [45], while the MPAO1 WT dispersed uniformly in the suspension.

### *FlgE* mutants exhibit reduced adhesion rate but increased biofilm growth under flow

**Fig. 4:**
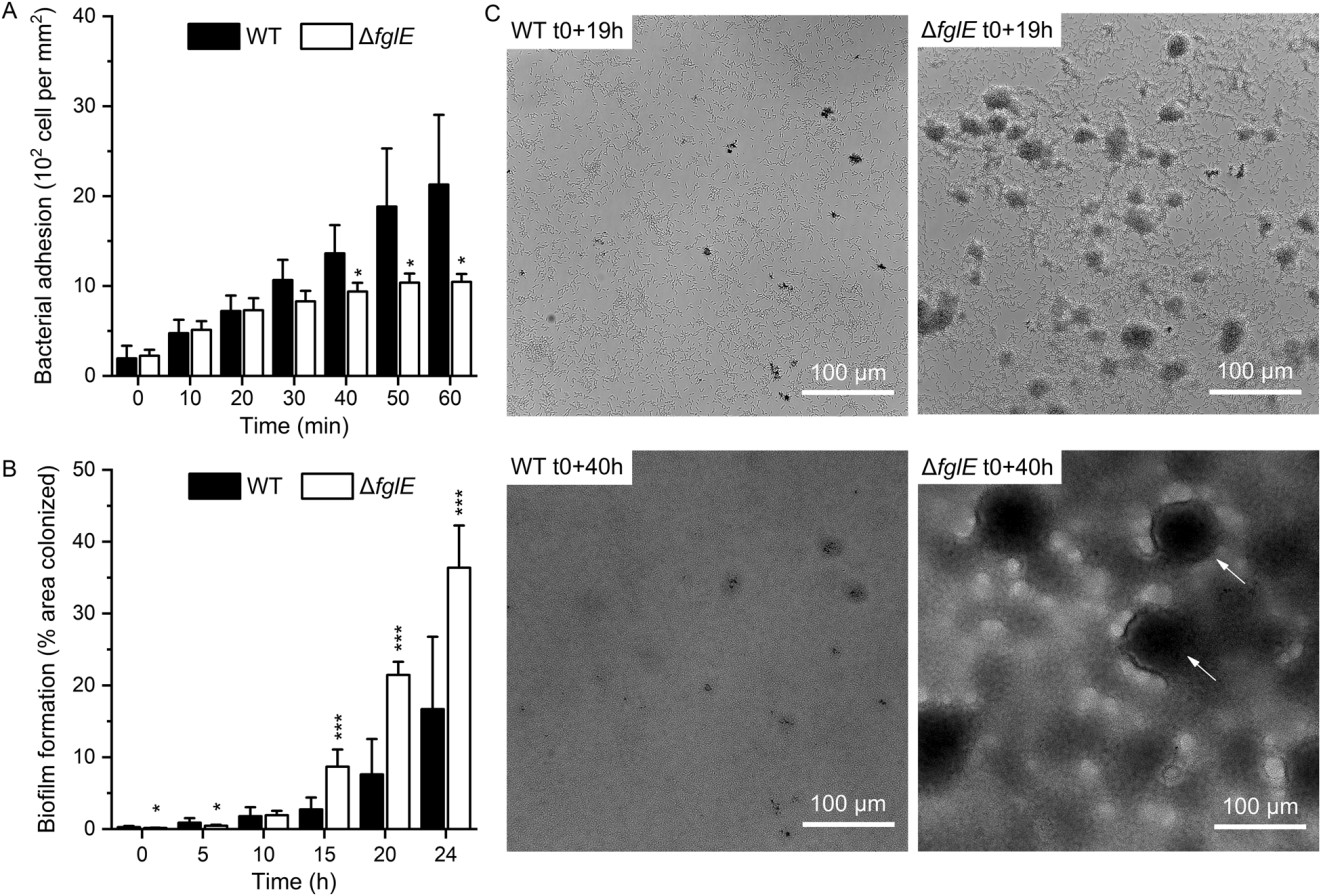
Dynamic bacterial adhesion and biofilm formation of MPAO1 WT and Δ*flgE* under flow conditions. **A** Bacterial suspensions were injected in a flow chamber with a shear rate of 65 s^−1^ for 1 h and the adhesion was quantified by counting adhering cells. **B** M9 medium was then injected in the flow cell and the biofilm growth was measured by the percentage of area colonized over 24 h. Results represent the mean ± SD of two biological repeats with **A** three and **B** six areas of observation per repeat. Student’s *t*-tests were performed in comparison to the MPAO1 WT with * equals to *P* < 0.05 and *** equals to *P* < 0.001. **C** Biofilm formed by MPAO1 WT and Δ*flgE* cells at the same position in the flow chamber after 19 and 40 h biofilm growth in M9 medium. t0 corresponds to the start of the biofilm growth phase, after the end of the bacterial injection phase. Arrows indicate the mushroom-like biofilm structures. Representative bright field images were selected from experiments performed in duplicates.

To investigate more thoroughly the dynamics of biofilm formation of the Δ*flgE* mutant and its antibiotic tolerance, the adhesion and biofilm growth of *P. aeruginosa* were studied under flow conditions in M9 medium. MPAO1 WT cells adhered faster than the Δ*flgE* mutant cells (Fig. 4A), but the adhering *flgE* cells displayed a faster growth rate than the MPAO1 WT strain (Fig. 4B). This striking observation is in contrast with the low biofilm biomass of the Δ*flgE* in microtiter plates. Additionally, under flow, the biofilm structure of the MPAO1 WT differed from that of the mutant (Fig. 4C). MPAO1 WT were spread across the whole surface and formed flat biofilms, while the Δ*flgE* mutant aggregated and grew biofilms preferentially as aggregates resembling mushroom-like structures. Movies of the growth of WT and Δ*flgE* biofilms are available in Movie S1 and S2.

### Biofilms of *flgE* mutant exhibit extreme tolerance to gentamicin under flow

**Fig. 5:**
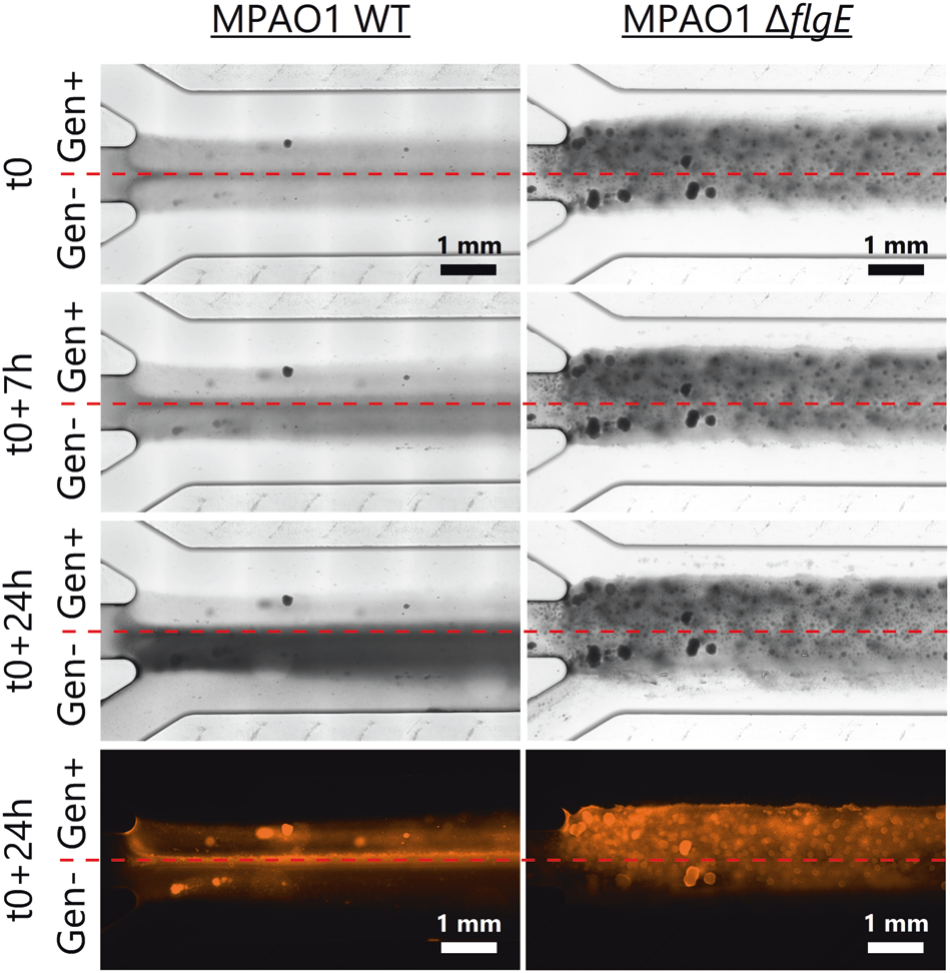
Bright field and fluorescence imaging of MPAO1 WT and *flgE* mutant during gentamicin treatment under flow conditions. Forty hours-old biofilms (t0) were exposed to M9 medium supplemented with 12 µg/mL of gentamicin (above red lines) and antibiotic-free M9 medium (below red lines). t0 corresponds to the start of gentamicin treatment, after 40 h of biofilm growth in M9 medium. After 24 h of treatment, dead (membrane damaged) bacteria were stained with PI and examined with a fluorescence microscope. The direction of the flow is from left to right. Overview images of the entire flow chamber after antibiotic treatment are available in Fig. S5.

Biofilms of the Δ*flgE* mutant were highly tolerant against antibiotics in microtiter plates assays and therefore we investigated their tolerance under flow. Forty hours-old biofilms were exposed to gentamicin at a concentration three times higher than the MBC (determined to be 4 µg/mL in Fig. 2). Upon gentamicin exposure, the *P. aeruginosa* MPAO1 WT biofilm became thinner while the Δ*flgE* biofilm remained largely unaffected (Fig. 5 and Fig. S5) and WT cells were removed from the biofilm while mutant cells stayed (Movie S3 and S4). Interestingly, MPAO1 WT biofilms grew much faster than Δ*flgE* biofilms under the untreated condition, suggesting a stagnation of the Δ*flgE* mutant biomass over time. SYTO9 and PI staining revealed that MPAO1 WT and Δ*flgE* biofilms exhibited higher PI intensity after gentamicin treatment than in the untreated condition (Fig. 5 and Fig. S6). Additionally, untreated WT biofilms exhibited a higher quantity of SYTO9-stained cells than the treated biofilms, whereas biofilms of the Δ*flgE* mutant exhibited the same level of SYTO9 staining in the untreated and treated conditions (Fig. S6). These results were confirmed by comparing MPAO1 WT and Δ*flgE* biofilms grown in the same flow chamber (Fig. S7). The PI staining of the cells grown in antibiotic-free M9 media was attributed to natural deaths within biofilms and staining of extracellular DNA. In general, these observations showed that *P. aeruginosa* cells missing *flgE* exhibit a unique mechanism of biofilm formation and an enhanced biofilm tolerance under a constant flow of gentamicin in comparison to the WT.

### *FlgE* mutants are protected from gentamicin in the inner part of the biofilm matrix

**Fig. 6:**
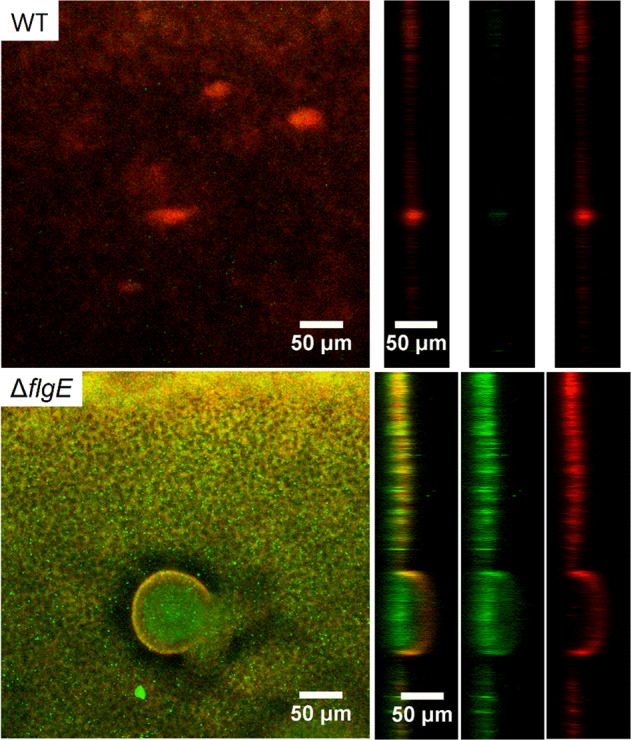
Confocal laser scanning microscopy observation of MPAO1 WT and Δ*flgE* biofilms after gentamicin treatment. Forty hours-old biofilms were exposed to M9 medium supplemented with 12 µg/mL of gentamicin for 24 h. SYTO9 and PI were used to stain living bacteria in green and membrane damaged bacteria in red, respectively. Orthogonal views of the mushroom-like biofilm are shown from left to right with an overlay of SYTO9 and PI, SYTO9 alone and PI fluorescence alone.

**Fig. 7:**
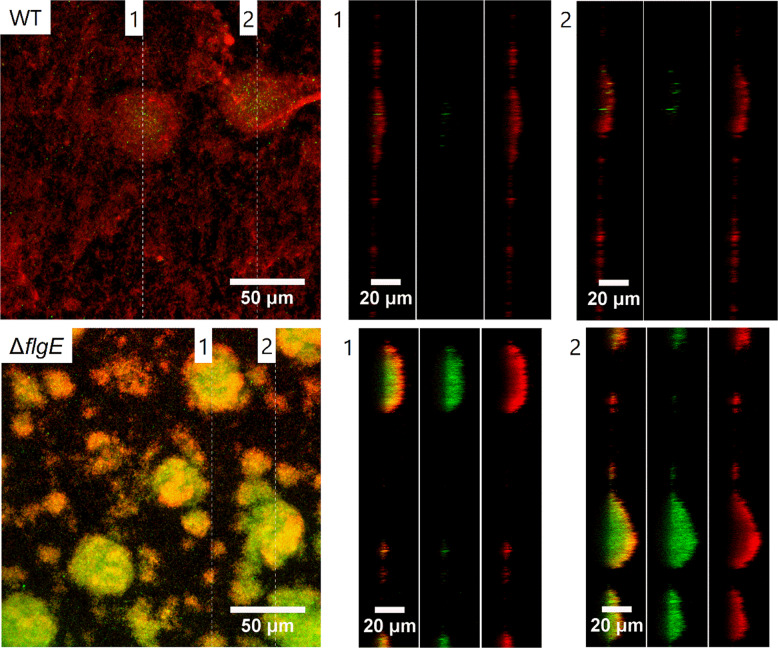
Gentamicin penetration through MPAO1 WT and Δ*flgE* biofilms observed by confocal laser scanning microscopy. Seventy-two hours-old biofilms were exposed to M9 medium supplemented with 2 µg/mL of TR-gentamicin for 20 min. Biofilms were then washed with 0.9% NaCl and living cells were stained with SYTO9. The green and red fluorescence correspond to the SYTO9 staining and the gentamicin, respectively. Two orthogonal views (labeled as “1” and “2”) are shown from left to right with an overlay of SYTO9 and TR-gentamicin, SYTO9 alone and TR-gentamicin fluorescence alone. Images are representative of two independent experiments.

To further analyze the proportion of dead (membrane damaged) cells within the mutant biofilms, confocal laser scanning microscopy was used to image *P. aeruginosa* MPAO1 WT and Δ*flgE* biofilms after 24 h gentamicin treatment and after staining with SYTO9 and PI. Biofilm cells of MPAO1 WT were greatly removed by the treatment, leaving only a thin layer of cells stained by PI (Fig. 6). In contrast, while the top layer of Δ*flgE* biofilm mostly contained membrane damaged bacteria, bacteria deep inside the biofilm structure were mostly viable. This observation suggests that gentamicin did not affect Δ*flgE* mutant cells located at the center of the biofilm, indicating that biofilm tolerance of Δ*flgE* biofilms is mediated by surviving cells deep inside the biofilm. To gain mechanistic understanding of the observed antibiotic tolerance, TR-gentamicin was used to measure the antibiotic penetration through the biofilm matrix (Fig. 7). The gentamicin levels at different depth of MPAO1 WT and Δ*flgE* biofilms were quantified and presented in the Supplementary Fig. S8. TR-gentamicin was found predominantly in the superficial layers of the Δ*flgE* biofilms, while the inner part of the biofilm did not contain gentamicin, which is in agreement with the relatively low cell killing in this region of the *ΔflgE* biofilm (Fig. 6). Under the same conditions, the entire biofilms of MPAO1 WT were found mostly red, demonstrating interaction with TR-gentamicin. These results suggest that the mechanism of tolerance observed in Δ*flgE* mutants is linked to an inhibition of antibiotic penetration.

### Genes relevant for the biofilm matrix are downregulated in *flgE* biofilm

**Fig. 8:**
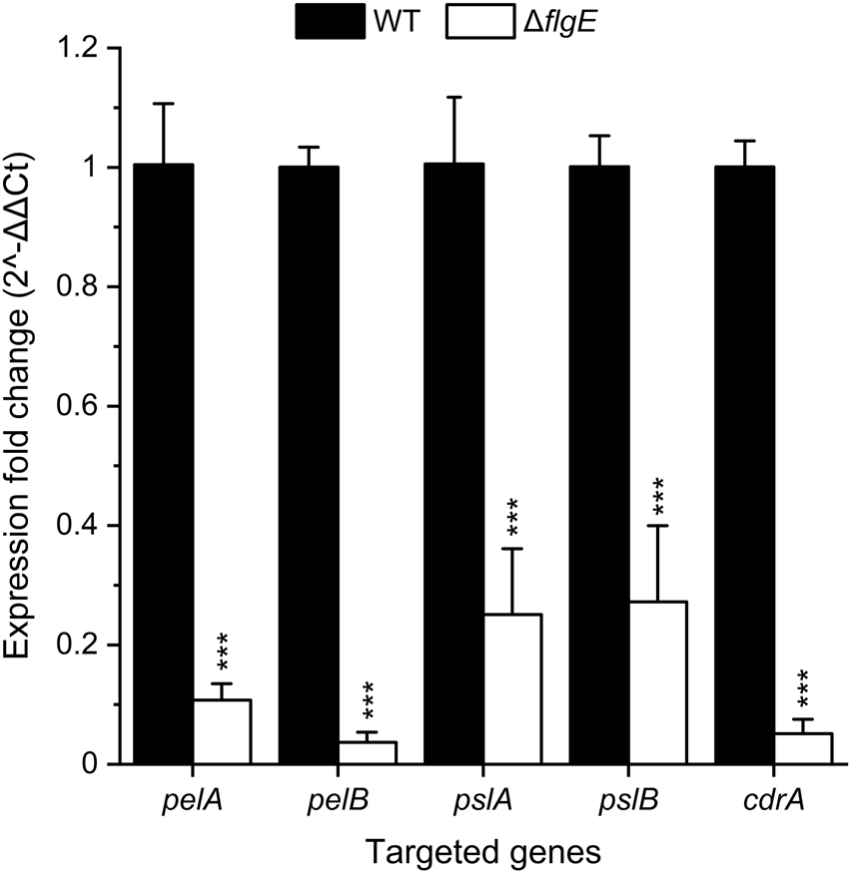
Fold change in mRNA levels of matrix genes in MPAO1 WT and Δ*flgE* biofilms. RNA was extracted from 24 h-old biofilms, grown in the same conditions as Fig. 2, and the expression of *rpsL*, *pelA*, *pelB*, *pslA*, *pslB*, and *cdrA* genes were quantified by qPCR. Expression fold change were normalized according to the expression of the housekeeping gene *rpsL* in MPAO1 WT (defined as 1) and in Δ*flgE* biofilms. Results represent the mean ± SD of three measurements done on two independent RNA extractions. Student’s *t*-tests were performed in comparison to the mRNA levels in MPAO1 WT with ***P < 0.001. All primers used are described in Table S1.

We further assessed whether knock-out of *flgE* can lead to differential expression of matrix genes, which might be involved in antibiotic penetration. Here, the expressions of *pelA*, *pelB, pslA*, *pslB*, and *cdrA* genes, which are well-known and important biofilm constituents, were quantified for biofilms formed by MPAO1 WT and Δ*flgE* mutant by qPCR. All matrix genes were found to be downregulated in the Δ*flgE* biofilms in comparison to the WT biofilms (Fig. 8). More specifically, the expressions of the *pelA* and *pelB* genes were reduced by 9 and 27 fold in Δ*flgE* biofilms, respectively. The *pslA* and *pslB* genes were both found to be downregulated by 4 fold, while the *cdrA* gene expression was reduced by 19 fold. Thus, these results strongly suggest that knock-out of *flgE* in *P. aeruginosa* alters the biofilm transcriptome, including genes encoding for the synthesis of known matrix components.

## Discussion

The flagellum of *P. aeruginosa* contributes greatly to bacterial motility and biofilm formation [46]. It also plays a major role in adhesion to surfaces, and especially increases adhesion to mucus in the lung of CF patients [47]. The flagellum is a virulence determinant, which triggers a strong inflammatory response [19]. In the case of CF infections, *P. aeruginosa* is found to repress the flagella synthesis and to select mutations leading to flagellar loss [20–23]. The goal of this study was to understand the role of the flagellar repression on biofilm physiology and to investigate the potential mechanism of flagellar-mediated antibiotic tolerance with a focus on the flagellar hook protein FlgE of *P. aeruginosa*. The flagellar hook of *P. aeruginosa* constitutes the basis of the flagellum and has been reported to be downregulated in biofilms cells [24, 25]. Furthermore, FlgE proteins were found to be absent from the membrane of *P. aeruginosa* grown in mucus [31].

Selected transposon mutants were screened to assess the influence of a given gene with respect to biofilm formation and biofilm tolerance. Our results demonstrated that inactivating the *flgE* gene altered the biofilm structure and dramatically increased the tolerance of *P. aeruginosa* to gentamicin. Interestingly, this effect was found to be specific to *flgE* mutants and not to *flgD* mutants (Fig. 1), which also plays a role in flagellum synthesis and is required for the assembly of a functional hook [26]. To avoid any polar effects caused by the transposon insertion, the *flgE* gene was removed by genetic knockout and proven to be perfectly in frame and scar-free (Fig. S2). The phenotype of the knockout mutant of *flgE* was similar to that of the transposon mutant of *flgE*, i.e., decreased biofilm biomass and increased biofilm tolerance (Fig. 2). Further characterizations revealed that *P. aeruginosa* missing *flgE* exhibited a loss of swimming ability, an enhanced growth yield and a reduced ability to adhere to abiotic surfaces (Figs. 3 and 4A and Fig. S4). The loss of swimming ability can be explained by the complete absence of flagellum in the *flgE* mutants, as shown by SEM observations (Supplementary Fig. S9). Inactivating *flgE* altered both the quantity and the morphology of the biofilm, but the effect was dependent of the growth conditions. After 24 h growth in microtiter plates, *flgE* mutants grew a thin biofilm layer on the walls of the well, while the MPAO1 WT formed additionally a thick biofilm layer at the air/liquid interface. This observation suggests that the non-motile cells of the *flgE* mutant grew as a biofilm wherever they adhered, whereas MPAO1 WT cells moved preferentially towards the higher concentration of oxygen and benefited from a better growth environment. In contrast, in a microfluidic flow chamber with a constant flow of nutrients, *flgE* mutants formed biofilms faster and thicker than the MPAO1 WT (Fig. 4B). As described in previous work, the biofilm formation is more dependent on bacterial motility in a static environment than in a flow chamber [48]. Consistent with other studies, *P. aeruginosa* WT formed rather flat biofilms likely due to its extensive surface motility [12, 17]. However, biofilms formed by *flgE* mutant exhibited patterns and a high amount of mushrooms-like structures (Fig. 4C).

Highly organized structures in biofilms have already been observed in *P. aeruginosa* [11–13]. Mushroom-like structures are known to increase biofilm cell tolerance and constitute a challenge for antibiotic treatment [6–8]. Although mainly explained by chemotaxis-related mechanisms [14], the emergence of these patterns remain unclear as recent models explain this phenomenon independently of chemotaxis [49]. Recently, Thomen et al. have demonstrated that biofilm patterns are dependent on physical forces of cells, where cells with high ability to self-assemble form highly organized biofilms, while low aggregative cells form unorganized and flat biofilms [50]. This can be one explanation for the observed difference of biofilm morphology for *flgE* mutant and MPAO1 WT cells under dynamic flow. Tolker–Nielsen’s group has suggested that the initiation of microcolonies is the first step towards mushroom-like structures and likely occurs through the clonal growth of non-motile cells [15, 18]. This is another explanation for the observed mushroom-like structure of the *flgE* mutant biofilm, due to its non-motility and high growth rate. Moreover, our study demonstrates that removing *flgE* increased the growth yield and the final biomass of *P. aeruginosa*, leading to a faster biofilm formation under constant refreshment of nutrients. Flagellum assembly and rotation constitutes a major metabolic cost and the repression of the pioneer protein of the flagellum, FlgE, can potentially lead to a metabolic advantage and a better allocation of resources [45, 51].

We also performed a preliminary study to assess whether the matrix genes are differently regulated in *flgE* biofilms, which could result in an altered biofilm formation. Our results demonstrate a reduction in the mRNA levels of genes involved in the production of biofilm matrix (i.e., *pelA*, *pelB*, *pslA*, *pslB*, and *cdrA*) in *flgE* biofilm in comparison to the WT biofilm after 24 h of culture (Fig. 8). These results are in contradiction with a recent work showing that flagellar mutants overproduced the biofilm matrix polysaccharides Psl and Pel in a surface-contact dependent manner [52]. This inconsistency could be caused by the different flagellum mutants and the experimental set up used between this and our study. Our results are coherent because the reduced expression of matrix genes in the *flgE* mutant fits with the low biofilm biomass observed in Fig. 2. Additionally, our observations are in agreement with the study of Staudinger et al., which demonstrated that mutants missing *flgK* and mutants inactivated in EPS production (*pelA*, *pslBCD*, and *algD*) were low biofilm formers, but displayed increased formation of aggregates in low density agar and increased tolerance to tobramycin [53]. To better understand the role of the *flgE* gene in biofilm structure and formation, it will be necessary to follow the expression of the matrix genes during the biofilm growth. While the underlying mechanisms remain to be identified, our results suggest that the repression of *flgE* and of flagellar synthesis reported in CF environment is likely a metabolic adaptation that not only increases *P. aeruginosa* fitness but additionally alters biofilm structure towards an aggregative and antibiotic-tolerant phenotype.

Indeed, our study shows that biofilms formed by *flgE*-deficient *P. aeruginosa* exhibited increased recovery after antibiotic treatment, suggesting a mechanism of antibiotic tolerance [54]. To rule out the impact of biofilm maturity on antibiotic tolerance, biofilms formed after various time periods have been investigated, namely 24 h (Figs. 2 and 4), 40 h, 40 + 7 h, 40 + 24 h (Figs. 5 and 6) and 72 h (Fig. 7). In all these conditions, the biofilms of the *flgE* mutant were consistently found to be more tolerant. In other words, no matter at which growth stage, the *flgE* mutant biofilm always showed higher tolerance than the WT, even at the rather early stage of 24 h when much less biofilm was formed by the mutant. The *flgE* mutant formed about 7-fold less biofilm than the WT after 24 h (Fig. 2A), however the formed mutant biofilm was already much more tolerant than that of the WT (Fig. 2B). Our results thus suggest that the enhanced antibiotic tolerance of the *flgE* mutant biofilm cannot be caused, at least largely, by the faster maturation of its biofilm, compared to the WT biofilm.

In comparison to *flgE*, inactivating the *flgD* gene, a scaffolding protein required for the initiation of flagellar hook assembly [55], did not lead to any increase in biofilm tolerance to gentamicin, thus proposing an important role of *flgE* in tolerance of *P. aeruginosa*. Planktonic cells of *flgE* mutants were found to be as sensitive to gentamicin, tobramycin, colistin, ciprofloxacin, and imipenem as MPAO1 WT (Fig. 2C), suggesting a biofilm-specific mechanism of tolerance for *flgE* mutants. Under constant nutrient flow, biofilms of the *flgE* mutant were thicker than those formed by the MPAO1 WT and were mostly unaffected by gentamicin at a concentration three times higher than the MBC-P, at which biofilms of the MPAO1 WT were eradicated (Fig. 5). Interestingly, biofilms of *flgE* mutants exhibited higher tolerance towards gentamicin than the MPAO1 WT independently of the total biofilm biomass.

These results are in agreement with the model of Landry et al., which reports that *P. aeruginosa* biofilms formed on mucin or *flgK*-deficient biofilms exhibit large cell aggregates and increased tolerance to tobramycin, hypothetically due to the limitation of antibiotics penetration [17]. The *flgK* gene (PA1086) encodes for a hook-associated protein that replaces the hook cap FlgD during the maturation of the hook FlgE in order to make the junction with the FliC proteins of the flagellar filament [26]. Although we did not observe an increased tolerance towards tobramycin in *flgE* mutant biofilms, we observed tolerance towards the other aminoglycoside, gentamicin. This difference might be explained by the different strains and experimental setups used. *P. aeruginosa* biofilms appear similar after inactivation of *flgK* or *flgE*, although our *flgE* mutant shows great twitching ability on glass surfaces (Movie S5 and S6) whereas *flgK* did not [17]. Transcriptomic studies on *P. aeruginosa* biofilms revealed downregulation of *flgE* and *flgD*, but not of *flgK* [24], suggesting a more direct role of *flgE* in the observed tolerance. Finally, by performing confocal laser scanning microscopic observations of Δ*flgE* biofilms after gentamicin treatment and live/dead staining, we demonstrate that only the superficial layer of biofilm cells was membrane damaged, while the inner part was unaffected by gentamicin (Fig. 6). To gain further understanding of this phenomenon, we used gentamicin conjugated with a fluorophore and confirmed that gentamicin was unable to penetrate into the inner parts of the *flgE* mutant biofilms (Figs. 7 and S8). Several studies demonstrated that the exopolysaccharides Pel and Psl are involved in the formation of microcolony structure and antibiotic resistance [56, 57]. Interestingly, we demonstrated that the *pslAB* and *pelAB* genes are downregulated by 4 and 9 to 27 fold in the *flgE* mutant biofilms in comparison to the WT, respectively (Fig. 8). As Psl plays a more important role than Pel in the survival of PAO1 strains [56, 57], we hypothesize that the *flgE* mutant might downregulate the expression of Pel genes, leading to a maximal impact of Psl, optimal cell growth with faster aggregate formation, and increased survival. However, this hypothesis is preliminary and the exact mechanism leading to reduction of antibiotic penetration should be investigated further. Further work is needed to characterize the dynamics of gene expression in biofilms formed by flagellum mutants. To this end, it might be especially relevant to perform transcriptomic studies at different stages of biofilm maturation.

In summary, these results suggest that the increased tolerance of *flgE* biofilms is due to a non-specific inhibition of antibiotic penetration. In addition, reduced growth, altered global gene expression or a decreased antibiotic activity at low oxygen concentrations in the inner part of the biofilm might also contribute to antibiotic tolerance in *ΔflgE* biofilms. Although the metabolic activity of cells at the inner part of the Δ*flgE* biofilm remains to be investigated, it is likely that this inner population is responsible for the re-colonization in fresh media after antibiotic treatment, as observed in Figs. 1B, C and 2B. Our study demonstrates for the first time the involvement of *flgE*, the flagellar hook protein, in increased biofilm tolerance of *P. aeruginosa* towards several antibiotics. Cells missing *flgE* self-assemble into a thick biofilm matrix due to their enhanced growth yield and biofilm growth. Consequently, the downregulation of *flgE* in *P. aeruginosa* biofilms and the absence of *flgE* in *P. aeruginosa* grown in CF mucus may represent an active mechanism of adaptation allowing the emergence of microcolony aggregates and an increased antibiotic tolerance of biofilm cells. As this mechanism does not occur in *P. aeruginosa* MPAO1 WT grown in vitro, using *flgE* mutants to mimic *P. aeruginosa* behavior in CF may be one key to improve our understanding of biofilm structure and to improve antibiotic treatments. While the involvement of *flgE* in antibiotic tolerance remains to be investigated for other motile bacterial pathogens, the knowledge gained in this work will help to understand the biofilm specific tolerance and facilitate development of counteracting strategies. In the future, it may be possible to develop a treatment approach to eradicate compact biofilm matrix before or when an appropriate antibiotic therapy is being administered.

## Supplementary information

Supplemental Materials

Video 1: Biofilm growth of MPAO1 WT in M9 medium

Video 2: Biofilm growth of ΔflgE mutant in M9 medium

Video 3:Biofilm of MPAO1 WT under gentamicin treatment

Video 4: Biofilm of ΔflgE mutant under gentamicin treatment

Video 5: Twitching of MPAO1 WT on glass

Video 6: Twitching of ΔflgE mutant on glass
