# Supplementary material for: Role of the flagellar hook in the structural development and antibiotic tolerance of Pseudomonas aeruginosa biofilms
Source: ISME J. 2021 Dec 8;16(4):1176–86. doi: 10.1038/s41396-021-01157-9 (PMC8940932; doi:10.1038/s41396-021-01157-9)
Supplement: Supplementary file 1 — Supplemental Materials [file 41396_2021_1157_MOESM1_ESM.docx]

Supporting Information

Role of the flagellar hook in the structural development and antibiotic tolerance of *Pseudomonas aeruginosa* biofilms

Jules D. P. Valentin,^1,2^ Hervé Straub,^1,3^ Franziska Pietsch,^4^ Marion Lemare,^1^ Christian H. Ahrens,^5^ Frank Schreiber,^4^ Jeremy S. Webb,^6^ Henny C. van der Mei,^2^ Qun Ren^1^*

^1^ Laboratory for Biointerfaces, Empa, the Swiss Federal Laboratories for Materials Science and Technology, St. Gallen 9014, Switzerland.

^2^ University of Groningen and University Medical Center Groningen, Department of Biomedical Engineering, Groningen 9712 CP, Netherlands.

^3^ Department of Plant and Microbial Biology, University of Zürich, Zürich 8008, Switzerland.

^4^ Federal Institute for Materials Research and Testing (BAM), Department of Materials and Environment, Division of Biodeterioration and Reference Organisms, Berlin 12489, Germany.

^5^ Agroscope, Research Group Molecular Diagnostics, Genomics & Bioinformatics and SIB Swiss Institute of Bioinformatics, Wädenswil 8820, Switzerland.

^6^ National Biofilms Innovation Centre, Institute for Life Sciences, University of Southampton, Southampton SO16 7 PX, UK

**** Correspondence:*** Qun Ren, Laboratory for Biointerfaces, Empa, Lerchenfeldstrasse 5, St Gallen 9014, Switzerland (+41 58 765 7688, qun.ren@empa.ch)

***Running Title:* Absence of FlgE promotes biofilm-specific antibiotic tolerance**

***Keywords*:** Cystic fibrosis, *flgE*, antibiotic resistance, microcolony aggregates, flagellar motility

**Table S1. Primers used in this study.**

| *Primers* | *Sequence (5'-3')* | *Amplified product* |
| --- | --- | --- |
| **Creation of knockout mutant** | | |
| *flgE*-frag1-Fw | gactctagaggatccccGCCGAGGGCGTCGATATG *^a^* | 5' of upstream of *flgE* |
| *flgE*-frag1-Rv | tccggcaaggagctatccTGACGGCGTGCCGGTG *^a^* | 3' of upstream of *flgE* |
| *flgE*-frag2-Fw | caccggcacgccgtcaGGATAGCTCCTTGCCGGA *^a^* | 5' of downstream of *flgE* |
| *flgE*-frag2-Rv | ttcgagctcggtacccCAGCACCGTGGAAGGC *^a^* | 3' of downstream of *flgE* |
| **qPCR analysis** | | |
| *rpsL*-Fw | GCTGTGCTCTTGCAGGTTGTG | *rpsL* |
| *rpsL*-Rv | GCAACTATCAACCAGCTGGTG |  |
| *pelA*-Fw | CCTTCAGCCATCCGTTCTTCT | *pelA* |
| *pelA*-Rv | TCGCGTACGAAGTCGACCTT |  |
| *pelB*-Fw | GACCCTGATCGATCTGCTGG | *pelB* |
| *pelB*-Rv | GCCAAGGATGTCCAGTTCCA |  |
| *pslA*-Fw | AAGATCAAGAAACGCGTGGAAT | *pslA* |
| *pslA*-Rv | TGTAGAGGTCGAACCACACCG |  |
| *pslB*-Fw | GCATGCCGAAACCCTTCA | *pslB* |
| *pslB*-Rv | GCGATACGCAGGAAGGTCTT |  |
| *cdrA*-Fw | ACGCCTACGTCAACAGTCAG | *cdrA* |
| *cdrA*-Rv | TTACCGGTGATCGCGTACTG |  |

^a^ Sequences in lower case are common sequences for overlap amplification with upstream and downstream fragment and with the vector pKmobsacB.


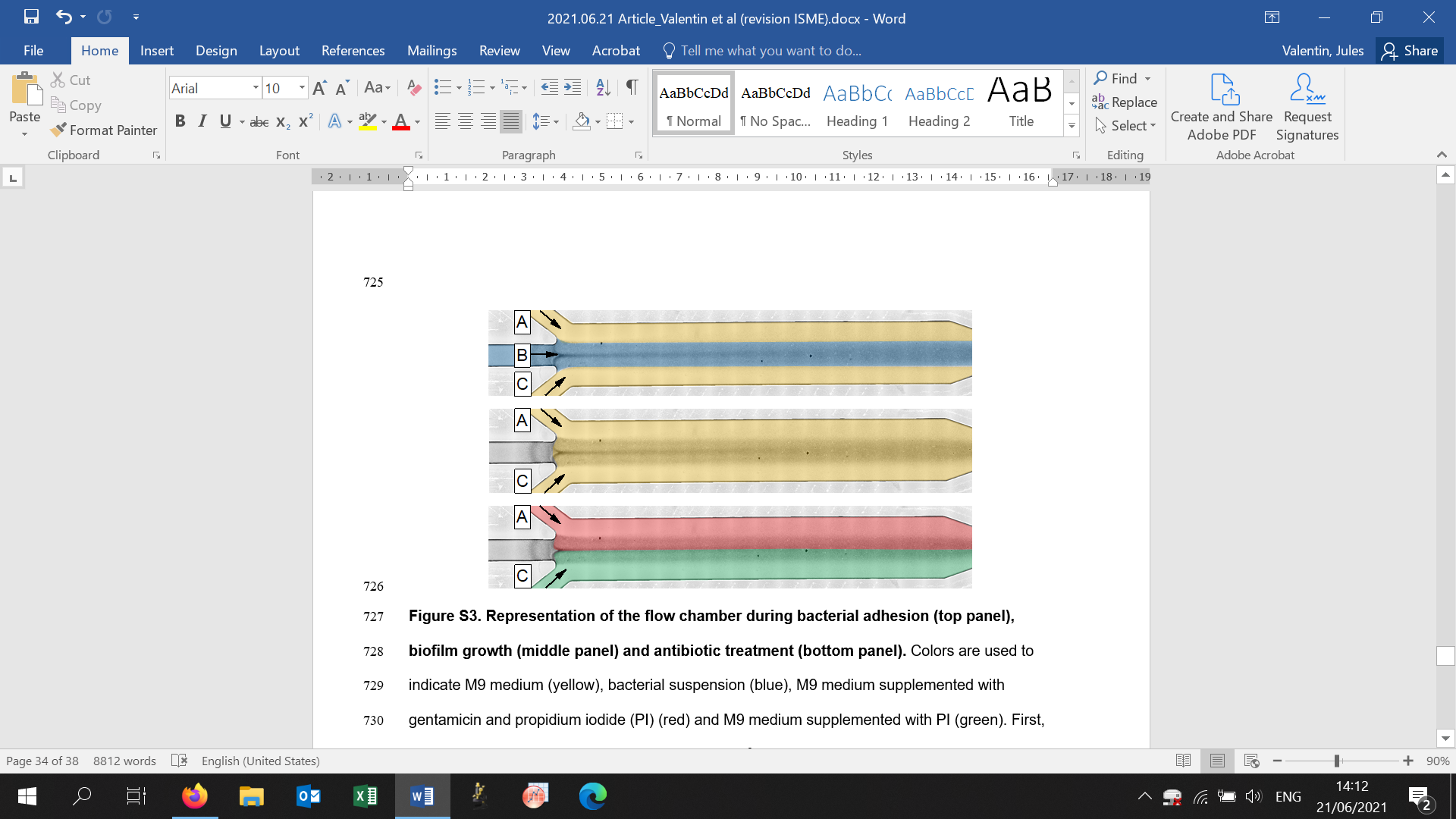


**Figure S1. Representation of the flow chamber during bacterial adhesion (top panel), biofilm growth (middle panel) and antibiotic treatment (bottom panel).** Colors are used to indicate M9 medium (yellow), bacterial suspension (blue), M9 medium supplemented with gentamicin and propidium iodide (PI) (red) and M9 medium supplemented with PI (green). First, a bacterial suspension at a concentration of 5 X 10^6^ CFU/mL is injected through channel B during 1 h at 200 µL/min. After 1 h, channel B is closed and sterile M9 medium is injected through channels A and C for 40 h at 200 µL/min. Finally, antibiotic treatment is performed by injecting M9 medium supplemented with 3 µM of PI and 12 µg/mL of gentamicin in channel A and M9 medium supplemented only with 3 µM of PI in channel C for 24 h at 200 µL/min. Flow direction is from left to right.

**Figure S2. Confirmation of *flgE* deletion in *P. aeruginosa* MPAO1 WT.** Primers were used to amplify the region from the 5' of the upstream to the 3' of the downstream region of *flgE*. All PCR analysis were performed using 3 µL of the bacterial suspension as DNA source, 200 µM of each nucleotides (N0447S, NEB, USA), 0.5 µM of each adequate primers, 3% DMSO, 1X Buffer Phusion HF and 20 U/mL of Phusion DNA Polymerase (Phusion High-Fidelity DNA Polymerase, M0530, NEB, USA). Cycling conditions were 95°C for 30 s, followed by 35 cycles of 95°C for 10 s, 63°C for 30 s and 72°C for 2 min, and a final extension at 72°C for 5 min. PCR products were separated by electrophoresis during 1 h at 100V in agar prepared at 1% (w/v) agarose (V3125, Promega, Spain) and the size of each amplicon was compared to a 1 kb DNA ladder (GeneRuler 1 kb DNA Ladder, SM0333, Thermo Scientific, USA). Amplicons of the deletion sites were extracted with a gel extraction kit (GeneJET Gel Extraction and DNA Clean Up, K0831, Lithuania) and sent for sequencing to Microsynth. 1. DNA Ladder 2. No DNA 3. *P. aeruginosa* MPAO1 WT 4. *E. coli* St18 containing the plasmid Pk19 with the upstream and downstream regions of *flgE* 5. Knockout mutant of MPAO1 missing the *flgE* gene. The sequencing of the deletion site of MPAO1 Δ*flgE* confirmed that the targeted gene was removed and that the upstream and downstream regions were bound together without any residual nucleotides.





**Figure S3. Assessment of biofilm tolerance to gentamicin by spotting *P. aeruginosa* MPAO1 WT and Δ*flgE* mutant complemented or not by a plasmid containing the *flgE* gene (p*flgE*).** 24 h-old biofilms were exposed to M9 medium supplemented with a gradient of gentamicin concentrations for 24 h and then allowed to recover in antibiotic-free M9 medium for 24 h. Five µL of biofilm suspensions were then spotted on BHI agar plates, followed by a 24 h incubation at 37°C. Three biological repeats with three technical repeats each were done and representative spotting images of one of the biological repeat are shown here. Complementation was performed by using the vector pMH4 PaCD00282523 containing the *flgE* gene and a resistance marker for ampicillin, purchased from DNASU plasmid repository. The vector was transformed into the Δ*flgE* mutants by electroporation and kept under constant pressure with ampicillin. To avoid the influence of ampicillin on bacterial physiology, complemented mutants were assessed for their tolerance to gentamicin in M9 medium supplemented or not with ampicillin.

**Figure S4. Growth curves of MPAO1 WT and Δ*flgE* mutant cells in M9 medium at 37°C in static conditions.** Overnight cultures of bacteria were diluted 1:100 in M9 medium and the turbidity at OD600 was measured every 30 min for 24 h to follow bacterial growth.


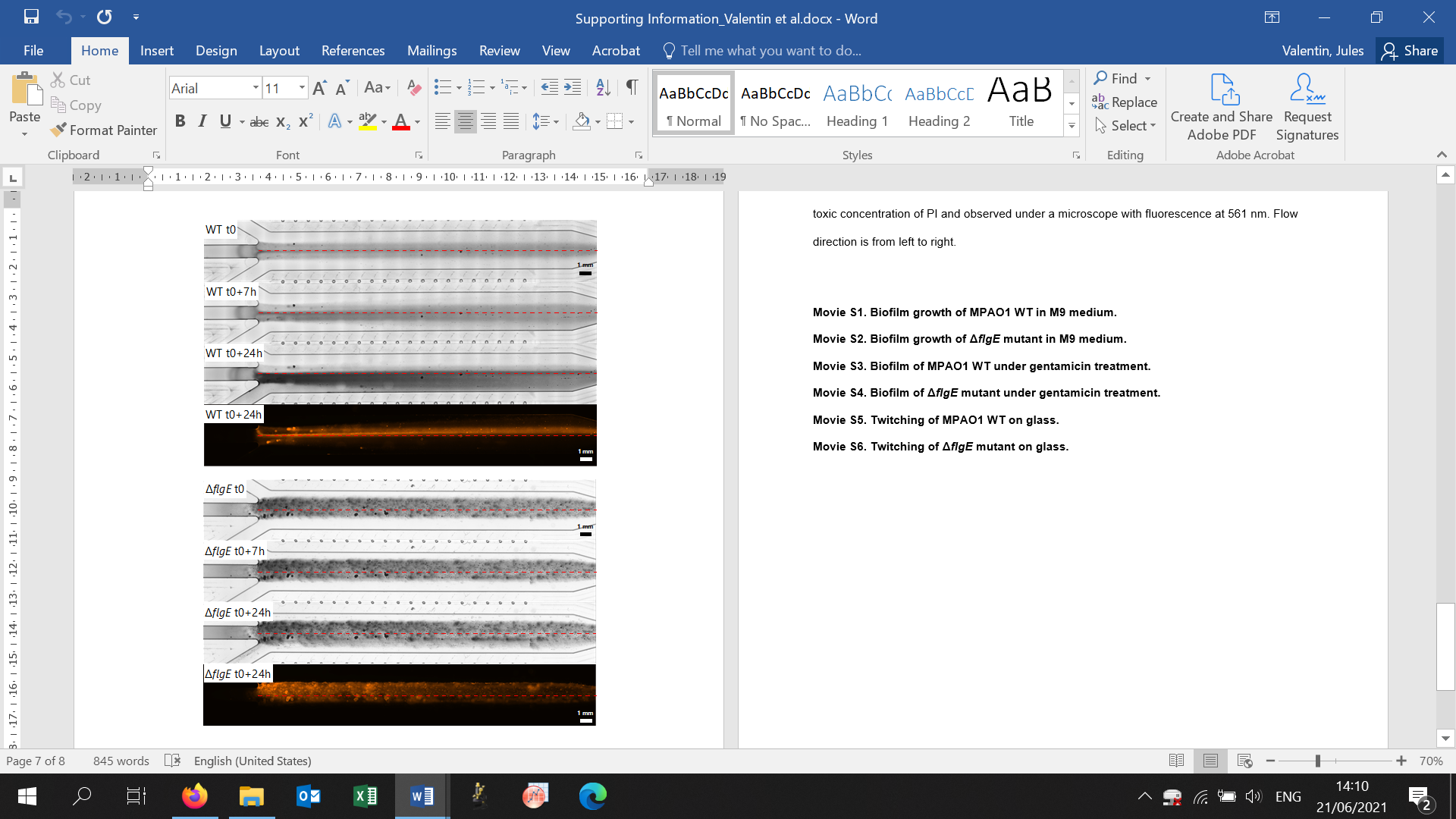


**Figure S5. Bright field and fluorescent observations of *P. aeruginosa* MPAO1 WT and Δ*flgE* biofilms during gentamicin treatment under flow.** 40 h-old biofilms were exposed to M9 medium supplemented with 12 µg/mL of gentamicin (above red lines) and antibiotic-free M9 medium (below red lines). t0 is corresponding to the start of gentamicin treatment, after 40 h biofilms growth in M9 medium. Dead (membrane damaged) bacteria were stained with a non-toxic concentration of PI and observed under a microscope with fluorescence at 561 nm. Flow direction is from left to right.

**Figure S6. Measure of the SYTO9 and PI fluorescence of (left) *P. aeruginosa* WT and (right) Δ*flgE* mutant biofilms after 24h gentamicin treatment.** Fluorescent intensities of SYTO9 and PI in the biofilms are quantified with ImageJ by measuring the mean gray values of 20 areas along the flow chamber.


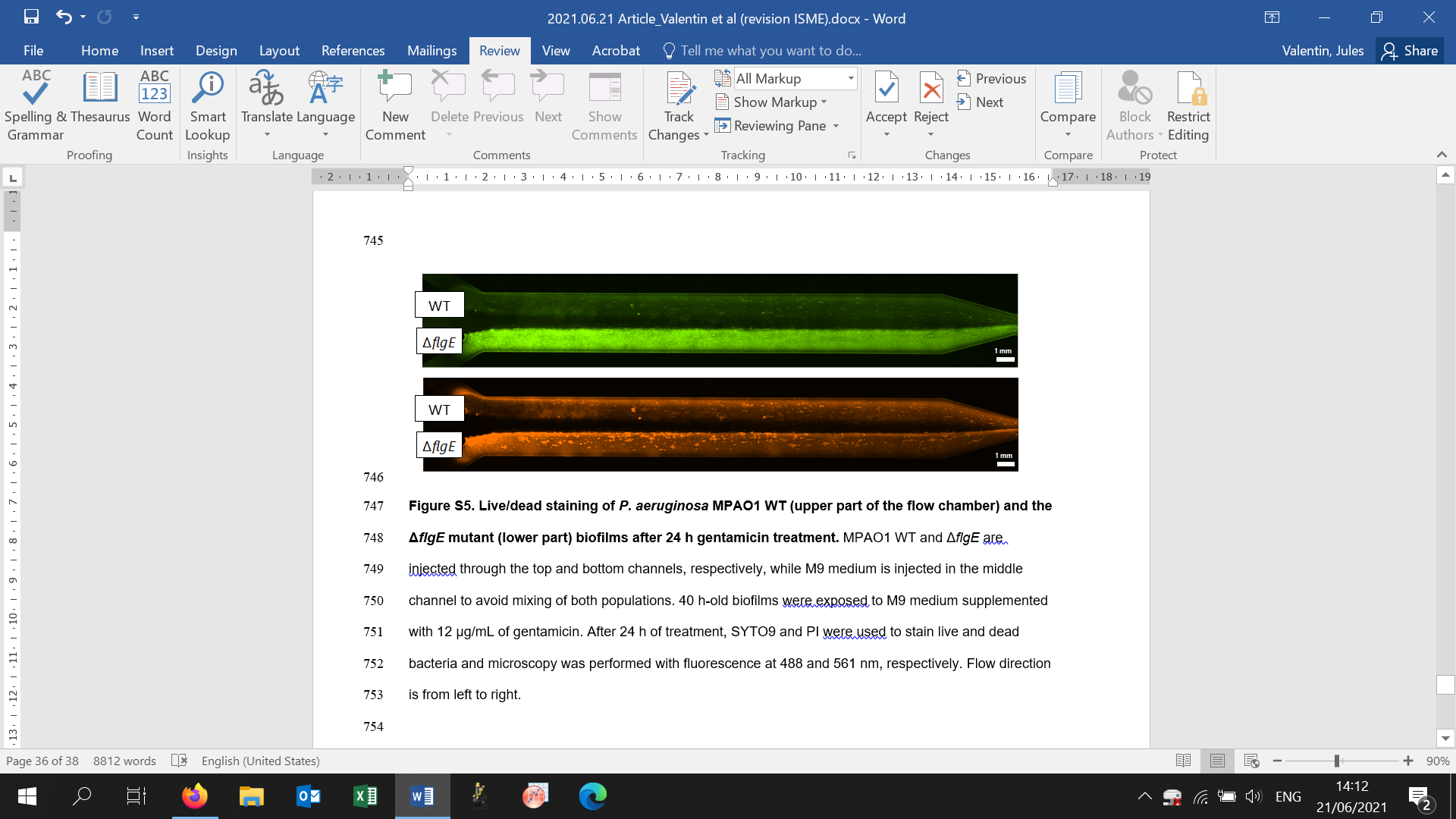


**Figure S7. Live/dead staining of *P. aeruginosa* MPAO1 WT (upper part of the flow chamber) and the Δ*flgE* mutant (lower part) biofilms after 24 h gentamicin treatment.** MPAO1 WT and Δ*flgE* are injected through the top and bottom channels, respectively, while M9 medium is injected in the middle channel to avoid mixing of both populations. 40 h-old biofilms were exposed to M9 medium supplemented with 12 µg/mL of gentamicin. After 24 h of treatment, SYTO9 and PI were used to stain live and dead bacteria and microscopy was performed with fluorescence at 488 and 561 nm, respectively. Flow direction is from left to right.

**Figure S8. Quantification of gentamicin presence in the different layers of a biofilm formed by MPAO1 WT (left) and Δ*flgE* (right).** Texas-Red gentamicin is quantified in the biofilms by measuring the fluorescence intensity using a confocal laser scanning microscope. The distance on the X-axes refer to a transversal cut of the biofilm aggregate, while the depth on the Y-axes represent the fluorescence signal measured at the different depth layers in the biofilm (0 being close to the surface). The results were obtained on one representative biofilm aggregate for the WT and the *flgE* mutant each.





**Figure S9. Observation of planktonic cells of *P. aeruginosa* MPAO1 WT and Δ*flgE* mutant by scanning electron microscopy (SEM).** Overnight cultures of *P. aeruginosa* prepared in BHI media were diluted 1:100 in M9 medium. Glass slides were immersed vertically in bacterial suspensions for 5 h at 37°C without shaking. Bacteria were then dried and fixed 4% paraformaldehyde (Sigma, Netherlands) and 2.5% glutaraldehyde (Sigma Aldrich, G5882, USA). Samples were sputtered to obtain a 10 nm gold layer (Leica, EM ACE600, Switzerland) and observed by SEM (Hitachi, S-4800, USA).

**Movie S1. Biofilm growth of MPAO1 WT in M9 medium.**

**Movie S2. Biofilm growth of Δ*flgE* mutant in M9 medium.**

**Movie S3. Biofilm of MPAO1 WT under gentamicin treatment.**

**Movie S4. Biofilm of Δ*flgE* mutant under gentamicin treatment.**

**Movie S5. Twitching of MPAO1 WT on glass.**

**Movie S6. Twitching of Δ*flgE* mutant on glass.**
